# Purification of mesenchymal stromal cell‐derived small extracellular vesicles using ultrafiltration

**DOI:** 10.1002/jex2.70030

**Published:** 2025-01-17

**Authors:** Rui Lei, Shuai Ren, Hua Ye, Zhanfeng Cui

**Affiliations:** ^1^ Institute of Biomedical Engineering, Department of Engineering Science University of Oxford Oxford UK

**Keywords:** diafiltration, membrane, mesenchymal stromal cells, small extracellular vesicles, ultrafiltration

## Abstract

Mesenchymal stromal cell‐derived small extracellular vesicles (MSC‐sEVs) are pivotal for the curative effects of mesenchymal stromal cells, but their translation into clinical products is hindered by the technical challenges of scaled production and purification. Ultrafiltration, a pressure‐driven membrane separation method, is well known as an efficient, scalable, and cost‐effective approach for bioseparation. However, there has been little study so far that comprehensively evaluates the potential application of ultrafiltration for scaled sEV isolation and purification. In this study, the feasibility and effectiveness of ultrafiltration for MSC‐sEV isolation and purification are studied, and the effects of key process design and operational parameters, including the membrane pore size, transmembrane pressure (TMP), stirring speed (shear rate), feed concentration, are quantified using a stirred cell setup. Results revealed that 500 kDa molecular weight cut‐off (MWCO) polyethersulfone membrane demonstrated superior suitability for MSC‐sEV separation, yielding higher purity and productivity compared to 100 and 300 kDa MWCO membranes of the same material. The MSC‐sEV productivity and purity could also be improved by applying a moderate stirring speed and lower operational pressure, respectively. Isovolumetric diafiltration was incorporated to enhance the purity of MSC‐sEVs, successfully removing about 99% of protein contaminants by six diafiltration volumes (DVs). Subsequently, a fed‐batch ultra‐diafiltration (UF/DF) process with optimised filtration parameters was developed and compared with the currently most used ultracentrifugation (UC) method, showing exceptional effectiveness and performance in the isolation of MSC‐sEVs: it increased the recovery of MSC‐sEV from 20.59% to 60.88% (about three folds increase) and nearly doubled the purity, while also reducing processing time from over 4 h to 3.5 h, with a potential further reduction to less than 2.5 h through automation. The study concludes that ultrafiltration could be a promising method for both lab‐scale preparation and industrial‐scale manufacture of MSC‐sEVs, offering advantages of high recovery, scalability, fast, and cost‐effectiveness.

## 1 INTRODUCTION

Small extracellular vesicles (sEVs) are nano‐sized bilipid membranous particles released by cells, ranging from 30 to 200 nm and usually with spherical shape (Szatanek et al., 2017; Théry et al., 2019). Due to their immunomodulating potency (Burrello et al., 2016), regenerative activity, systematic distribution and specific targeting ability (Vader et al., 2016), sEVs have been broadly reported to have a huge potential as a therapeutic strategy for various diseases and tissue regeneration, for example in skin (Nguyen et al., 2024), adipose (Zhao et al., 2018), kidney (Zhong et al., 2022), liver (Zhang et al., 2023), heart (van de Wakker et al., 2024), bone (Jia et al., 2019), cartilage (Kouroupis et al., 2023). Particularly, sEVs derived from mesenchymal stromal/stem cells (MSC‐sEVs) have emerged as key paracrine mediators that induce the curative functions of mesenchymal stromal/stem cells (MSCs) and therefore can serve as a critical cell‐free therapeutic tool in regenerative medicine, without introducing safety issues associated with live cell‐based treatment (Herberts et al., 2011).

Robust isolation and purification of MSC‐sEVs from MSC conditioned medium (CM), which is a complex mixture of cell secretome with high heterogeneity (Dowling & Clynes, 2011), is a key technical requirement to ensure the application of high‐quality MSC‐sEVs with consistent properties, purity and yield for the downstream therapy (Grangier et al., 2021; Syromiatnikova et al., 2022). In addition, to meet the high demand for MSC‐sEVs in their clinical translation and commercialisation, scalability is an essential characteristic of any practical separation and purification methods. Ultracentrifugation (UC) is currently the most commonly used approach for laboratory scale sEV separation, which allows the isolation process from about several hundred conditioned medium (Royo et al., 2020). However, UC suffers the obvious drawbacks of co‐isolation of the contaminants, mechanical damage for sEVs due to high shear force (Linares et al., 2015), poor accessibility of the ultracentrifuges and rotors owing to their high cost, and labour intensive (Xu et al., 2016). The commercial rotor‐dependant working capacity and the batch process also largely limit the scalability of the UC method (Ng et al., 2019; Watson et al., 2018). Other separation methods including precipitation (Brownlee et al., 2014), affinity‐based capture (Nakai et al., 2016), and size exclusion chromatography (Böing et al., 2014), have also been studied in the previous sEV research, while limitations regarding the introduction of undesirable precipitating reagents, high expenses, and the small working volume hamper the application of above methods in large scale isolation of sEVs for clinical purpose, respectively (Phan et al., 2018).

Recently, membrane filtration, especially ultrafiltration, a pressure‐driven membrane separation method that is widely used in purification of bioproducts such as antibodies and enzymes, has been proposed as an sEV isolation method due to its high scalability and affordable costs, which also makes it applicable for the industrial scale sEV manufacture (Syromiatnikova et al., 2022). Indeed, a seven‐fold yield of MSC‐sEVs was demonstrated by Haraszti et al. using tangential flow filtration with 500 kDa molecular weight cut‐off (MWCO) hollow fibre filter, compared to the traditional UC method (Haraszti et al., 2018). Heinemann et al. also reported that sEVs with satisfied purity and size distribution could be obtained by their self‐developed sequential filtration protocol consisting of dead‐end filtration with 0.1 µm polyethersulfone membrane, tangential flow filtration by 500 kDa MWCO hollow fibre filter, and final filtration with 100 nm track etch filter (Heinemann et al., 2014). Increasing applications of membrane filtration method in the field has been reported. However, little is known about sEV‐membrane interaction and the influence of various ultrafiltration parameters on the recovery, purity and productivity of sEVs, which are important to the development and optimisation of ultrafiltration‐based sEV isolation protocol.

In this study, the effects of different ultrafiltration parameters including membrane pore size, stirring speed, operational pressure, feed concentration and diafiltration on the isolation of sEVs were systematically studied, with the aim of retaining most of the sEV particles (yield), maximising the removal the non‐sEV protein contaminants (purity), and meanwhile achieving higher sEV productivity. In the experiments, 20 mL conditioned medium (also termed as feed) was concentrated to 2 mL (retentate) through the ultrafiltration membrane within a stirred cell membrane filtration system, and the permeate flow rate was recorded in the meantime. The feed, the permeate and the retentate were collected and analysed in terms of particle number, particle size distribution and protein amount. The recovery of the particle, the transmission of the protein contaminants, the purity and the productivity of the sEVs were subsequently calculated to evaluate the purification of sEVs. Based on the test results, a fed‐batch ultra‐diafiltration (UF/DF) protocol with optimised filtration parameters was established for the isolation and purification of MSC‐sEVs. The sEV isolated by this method was compared with that of the traditional UC method, regarding the processing time, sEV size distribution, yield, purity, biomarker expression and morphology. Our research is the first study that comprehensively explores the influence of different ultrafiltration parameters on the purification of sEVs. Such a fed‐batch UF/DF process showed outstanding advantages for both lab‐scale preparation and industrial‐scale purification of MSC‐sEVs.

## 2 MATERIALS AND METHODS

### 2.1 Preparations

#### 2.1.1 Preparation of EV‐depleted medium

The Fetal Bovine Serum (FBS, Cat.: 10270106, Gibco, USA) was ultracentrifuged for 18 h at 120,000 × *g*, 4°C using Optima XPN‐80 Ultracentrifuge (Beckman Coulter, USA) with SW 32 Ti Rotor (Beckman Coulter, USA) to deplete autologous EVs. The supernatant, regarded as EV‐depleted FBS, was collected and sterilised by filtering through a 0.22 µm vacuum filter apparatus (Cat.: 431097, Corning, USA). The EV‐depleted medium was made up of Dulbecco's Modified Eagle Medium (DMEM, Cat.: 31885049, Gibco, USA), 10% sterilised EV‐depleted FBS and 1% Penicillin‐streptomycin (Cat.: 15140122, Gibco, USA).

#### 2.1.2 Cell cultivation

The human telomerase reverse transcriptase‐immortalized bone marrow mesenchymal stromal cells (hTERT‐MSCs) at passage 22 were cultured at a seeding density of 2.5 × 10^6^ cells in 25 mL complete medium (DMEM with 10% FBS and 1% Penicillin‐streptomycin) per T175 flask (Cat.: 10296861, Corning, USA) at 37°C, 5% CO_2_. After overnight cell attachment, the medium was discarded and replaced with 25 mL fresh EV‐depleted medium in each T175 flask. The crude conditioned medium was collected after 48 h.

#### 2.1.3 Preparation of conditioned medium

The crude conditioned medium was centrifuged at 2000 × *g* for 15 min by Labofuge 400R Centrifuge (Heraeus, Germany) to remove the dead cells and cell debris, and then centrifuged at 10,000 × *g* for 40 min by Eppendorf centrifuge 5804R (Eppendorf, Germany), of which the supernatant was collected for later sEV isolation experiments.

### 2.2 Ultrafiltration method for sEV isolation

#### 2.2.1 Ultrafiltration system

**FIGURE 1:**
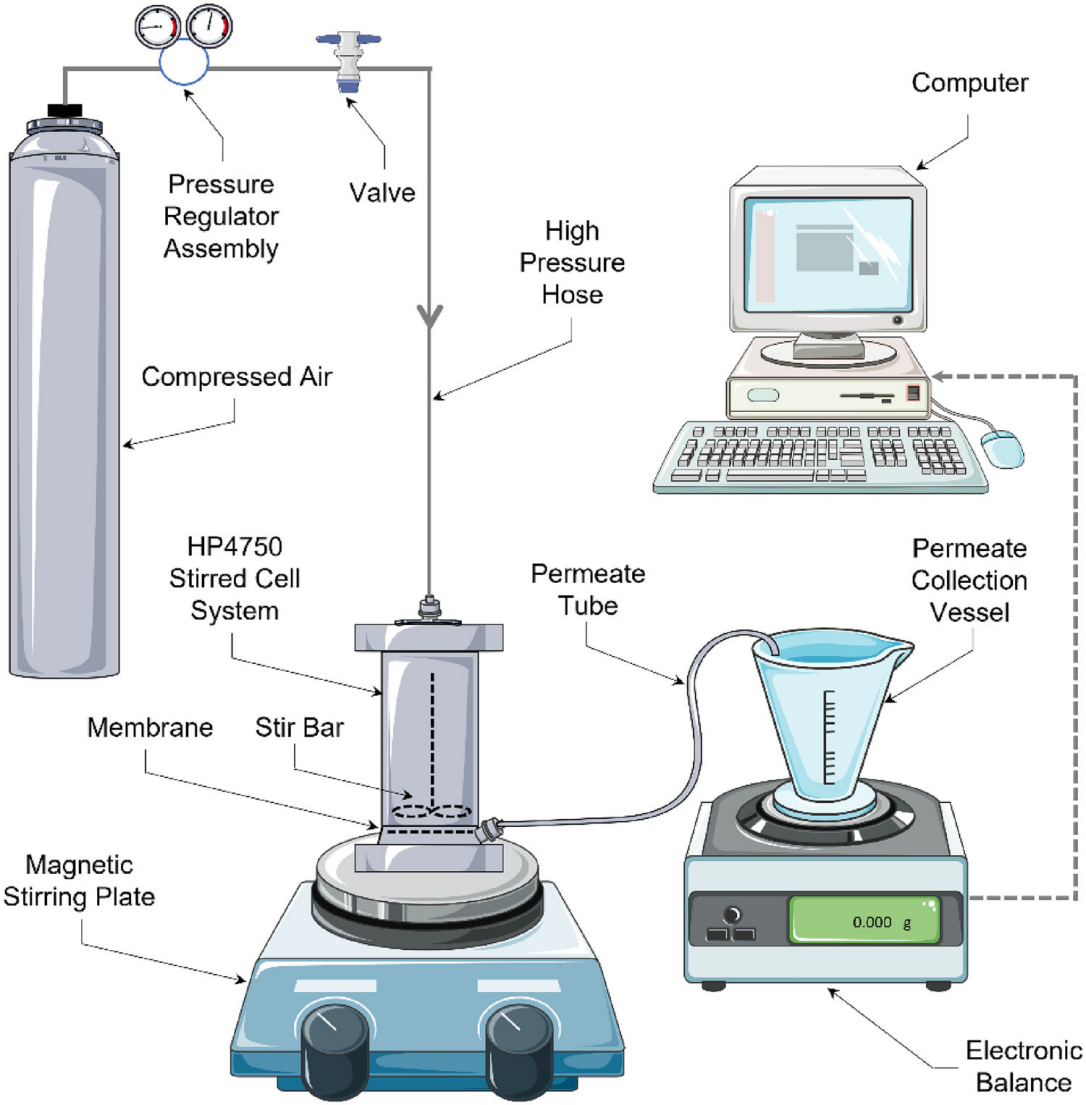
Experimental set‐up for stirred cell ultrafiltration system.

The HP4750 stirred cell (Sterlitech, USA) was utilized to conduct ultrafiltration for sEV isolation (Figure 1). The polyethersulfone membrane discs from Millipore, with a diameter of 4.7 cm and surface area of 17.35 cm^2^, were mounted at the bottom of this stirred cell for bioseparation. The operational pressure was provided by the compressed air and controlled by the pressure regulator and the valve. Stirring speed was applied by the stir bar using the IKA RCT basic magnetic stirrer (IKA, Germany). The permeate would go through the permeate tube and be collected in the permeate collection vessel on the Kern KB 2000–2 N precision balance (Kern, Germany), and the mass of the permeate was constantly recorded by the software RsWeight Ver.5.40 during the whole ultrafiltration process and can then be converted to the volume of the permeate.

#### 2.2.2 Membrane ultrafiltration test for sEV isolation

The membrane disc was firstly flushed with 250 mL UltraPure Distilled Water (Cat.: 10977035, Thermo Fisher Scientific, USA). The stirred cell was then filled with 20 mL conditioned medium, and the ultrafiltration was conducted under preset pressure and stirring speed. The ultrafiltration process was completed when 18 mL permeate was collected, that is, 2 mL retentate was obtained (10‐fold concentration by volume). The feed, retentate and permeate were collected and analysed after the filtration experiment. The influence of membrane pore size, stirring speed, applied pressure, feed concentration (direct dilution) and diafiltration process on sEV separation were examined. Membrane discs with 100, 300 and 500 kDa (Cat.: PBHK06210, PBMK06210, PBVK06210, Merck Millipore, USA) MWCO were cut on demand and used to study the effects of the membrane pore size. Stirring speeds ranged from 100 to 400 rpm and operational pressures from 5 to 25 psi were also tested. For the study of feed concentrations, 1× means no dilution process applied, 0.5× means that the feed is 20 mL conditioned medium diluted by 20 mL phosphate‐buffered saline (PBS), 0.25× means that the feed is 20 mL conditioned medium diluted by 60 mL PBS. For the study of the diafiltration process, 20 mL conditioned medium was firstly concentrated to 10 mL, followed by diafiltration including a dilution step with an equal amount (10 mL) of PBS and a concentration step to the half volume (10 mL), which is recorded as one diafiltration volume (DV). DV0 means no diafiltration process applied, DV2 means two times such diafiltration step applied, and DV6 means six times such diafiltration step applied.

#### 2.2.3 Fed‐batch UF/DF protocol for sEV isolation

This method was developed based on the setting shown in Figure 1. Totally 100 mL conditioned medium was fed to the system in several batches, with a concentration and diafiltration step in between. Specifically, 20 mL conditioned medium was firstly concentrated to 10 mL by 500 kDa MWCO membrane, followed by one diafiltration step (described in the previous section), a feed step of adding 10 mL conditioned medium and another concentration step to half volume, of which the last three steps together were regarded as one fed‐batch. After 8 times of fed‐batch, the 100 mL conditioned medium was concentrated to 10 mL. Seven times of isovolumetric diafiltration were then applied to the concentrated condition medium, and the retentate was concentrated to 700 µL at the end of the process, which was the sEV preparation obtained by the fed‐batch UF/DF method (please find Figure 6 for details).

### 2.3 UC method for sEV isolation

100 mL conditioned medium was centrifuged at 100,000 × *g* and 4°C for 90 min using Optima XPN‐80 Ultracentrifuge with 70 Ti rotor (Beckman Coulter, USA). The crude pellets were collected and then washed with PBS (Cat.: 10010015, Gibco, USA) using Optima MAX‐XP Ultracentrifuge (Beckman Coulter, USA) and TLA‐55 rotor (Beckman Coulter, USA) at 100,000 × *g* and 4°C for 75 min. The pellets obtained were resuspended in 100 µL PBS.

### 2.4 Characterisation and analysis

#### 2.4.1 Nanoparticle tracking analysis (NTA)

Particle movements were recorded for 60 s and repeated five times for each sample by NanoSight NS300 (Malvern Panalytical Ltd, UK) at camera level 13. sEV concentrations and size distributions were then analysed using NanoSight NTA 3.3 software (Malvern Panalytical Ltd, UK) at detect threshold 5.

#### 2.4.2 Protein extraction and quantification

The hTERT‐MSCs (passage 22) were trypsinised with 0.05% trypsin‐EDTA solution (Cat.: 25300062, Gibco, USA) and the cell pellet was obtained after 300 × *g* centrifugation for 5 min. Pierce RIPA buffer (Cat.: 89901, Thermo Fisher Scientific, USA) with cOmplete, Mini, EDTA‐free Protease Inhibitor (Cat.: 04693159001, Roche, Switzerland) were mixed with the MSC pellet. The mixture was incubated on ice for 30 min, followed by 20‐min centrifugation at 12000 rpm, 4°C using Eppendorf 5424R Centrifuge (Eppendorf, Germany). The supernatant was taken as MSC‐lysate. Feed (conditioned medium), retentate, permeate from membrane ultrafiltration tests as well as sEV preparations from both UC method and fed‐batch UF/DF method were lysed by adding RIPA buffer with freshly added protease inhibitor and incubating on ice for 30 min. QuantiPro BCA Assay Kit (Cat.: QPBCA‐1KT, Sigma‐Aldrich, USA) and SpectraMax i3x Microplate Reader (Molecular Devices, USA) were used to quantify the protein concentration.

#### 2.4.3 Western blot

Based on the previous protein quantification results, the protein concentration of cell lysate and sEV lysates were normalised to the same concentration by PBS. 4× Laemmli sample buffer (Cat.: 1610747, Bio‐Rad, USA) was added to all the samples, and NuPAGE sample reducing agent (Cat.: NP0004, Thermo Fisher Scientific, USA) was also added to the samples except those for CD63 detection. The mixture was boiled for 5 min at 95°C. Precision Plus Protein Kaleidoscope Prestained Protein Standard (Cat.: 1610375, Bio‐Rad, USA) and all the samples were electrophoresed on 4–20% Mini‐PROTEAN TGX Precast Protein Gels (Cat.: 4561094, Bio‐Rad, USA) for 95 min in Pierce Tris‐Glycine SDS Buffer (Cat.: 28362, Thermo Fisher Scientific, USA) at 120 V using Thermo EC135‐90 Electrophoresis Power Supply (Thermo Electron Corporation, USA). The proteins were transferred to Immun‐Blot PVDF Membrane (Cat.: 1620177, Bio‐Rad, USA) in Pierce Tris‐Glycine Buffer (Cat.: 28363, Thermo Fisher Scientific, USA) at 100 V for 90 min. After 5‐min wash in Pierce Modified Dulbecco's PBS Tween 20 Buffer (Cat.: 28346, Thermo Fisher Scientific, USA), the membranes were blocked with EveryBlot Blocking Buffer (Cat.: 12010020, Bio‐Rad, USA) for 30 min at room temperature. The membranes were probed with anti‐CD63 antibodies (1:1000; Cat.: ab134045, Abcam, UK), anti‐TSG101 antibodies (1:1000, ab125011, Abcam, UK) and anti‐GM130 antibodies (1:1000; Cat.: 610822, BD Biosciences, USA) at 4°C overnight, washed three times with PBS Tween for 5 min each time, and then incubated with secondary HRP‐linked Anti‐mouse IgG Antibody (1:1000; Cat.: 7076P2, Cell Signaling Technology, USA) or HRP‐linked Anti‐rabbit IgG Antibody (1:1000; Cat.: 7074P2, Cell Signaling Technology, USA) for 60 min at room temperature. After another three times wash in PBS Tween, Clarity Western ECL Substrate (Cat.: 170–5060, Bio‐Rad, USA) was applied to the membranes for 5 min at room temperature. The results were then imaged by Bio‐Rad Chemidoc MP System (Bio‐Rad, USA).

#### 2.4.4 Transmission electron microscopy (TEM)

Transmission electron microscopy was used to visualize the morphology of sEVs. A 10 µL sEV sample was added to freshly glow‐discharged 300 mesh carbon‐coated copper grids, followed by 10‐s negative staining using 2% uranyl acetate. Grids were air‐dried and imaged in a FEI Tecnai 12 transmission electron microscope at 120 kV using a Gatan OneView CMOS camera.

#### 2.4.5 Statistical analysis

Each experiment was repeated at least three times. Data were expressed as mean ± standard deviation (SD). Statistical analyses were performed by GraphPad Prism, version 8. Differences between the two groups were analysed by a tailed *t*‐test. Differences between three or more groups were analysed using one‐way ANOVA or two‐way ANOVA, followed by the Tukey method for multiple comparisons. The probability value lower than 0.05 was considered significant: *p ≤ 0.05, **p ≤ 0.01, ***p ≤ 0.001. Graphs were created by GraphPad Prism, version 8.

## 3 RESULTS

### 3.1 Effects of membrane pore size

As membrane filtration is a size‐based separation method, it is of great importance to choose a membrane with a suitable pore size for sEV separation. Membranes with different MWCO values of 100, 300 and 500 kDa were tested, and their performances on particle recovery, removal of protein contaminants, the purity of sEV preparations, permeate flux, and productivity were evaluated.

**FIGURE 2:**
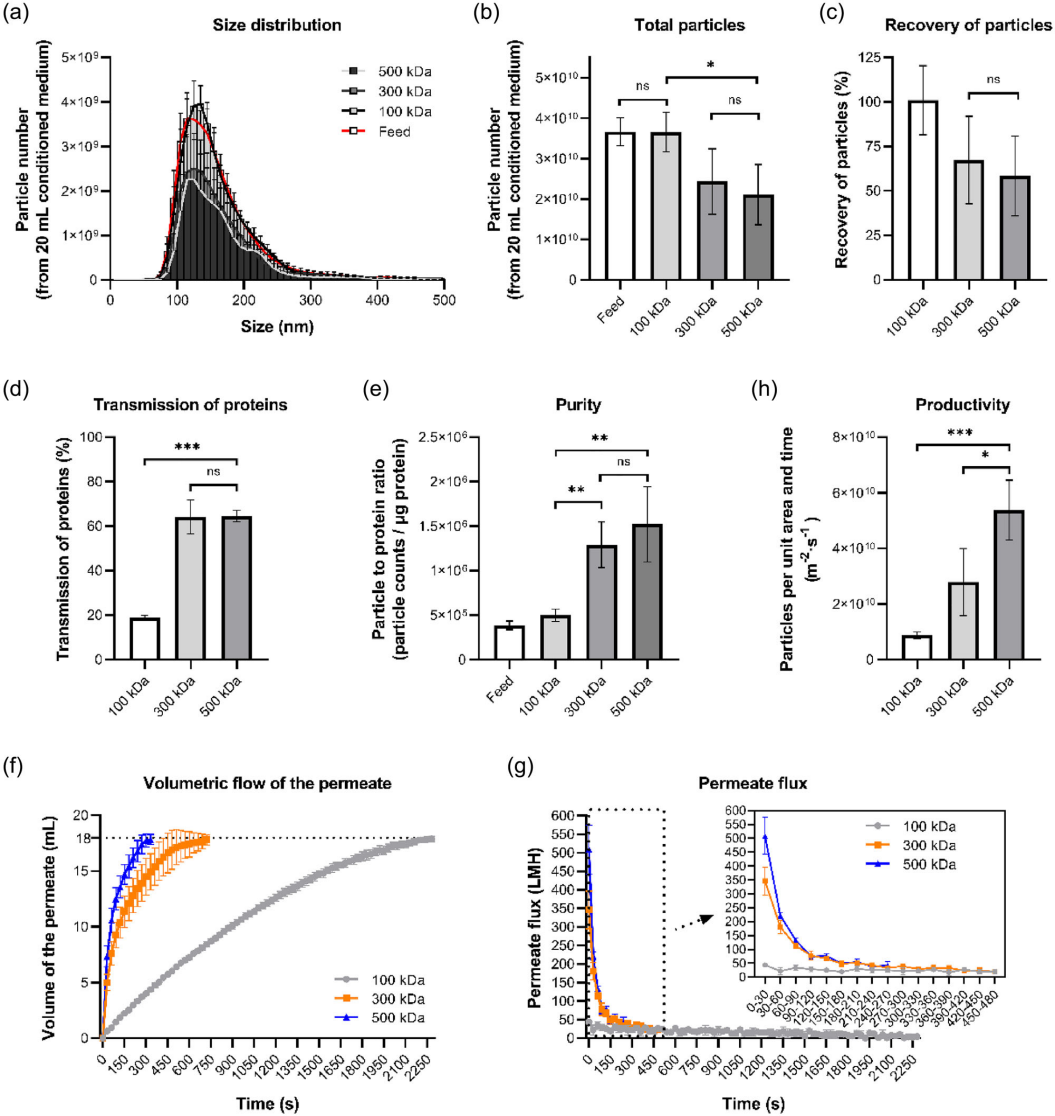
The effects of membrane pore size on sEV isolation, under 200 rpm stirring speed and 10 psi applied pressure. (a) Particle size distributions of the feed solution (CM) and the sEV preparations isolated by different MWCO membranes. (b) Total particle numbers of CM and the sEV preparations. (c) Recovery of particles from CM by different MWCO membranes. (d) Transmission of contaminated proteins during the ultrafiltration using different MWCO membranes. (e) Purity (particle to protein ratio) of CM and the sEV preparations. (f) Volumetric flow rate of the permeate during the ultrafiltration using different MWCO membranes. (g) Permeate flux during the ultrafiltration using different MWCO membranes. (h) The average productivity of sEVs using different MWCO membranes. CM, conditioned medium; MWCO, molecular weight cut‐off; sEVs, small extracellular vesicles.

The size distribution of the nanoparticles retained by three different membrane filters was similar and corresponded to that of the feed and also the characteristic profiles of EV preparations, with the majority falling within the size range of 30 to 200 nm (Figure 2a). The recovery of particles was defined as the ratio of the total particle number of the retentate to that of the feed. As expected, the 100 kDa membrane showed the highest recovery of particles as it has the smallest pore size amongst the three. A significantly larger number of particles, nearly 100% of the particles from the feed, was recovered by the 100 kDa membrane, while both 300 and 500 kDa filters showed about 40% loss of the nanoparticles, with no significant difference between the two (Figure 2b, c). Surprisingly, the number of particles in the permeates of these two membranes only occupied less than 4% of the total particles in the feed (data not shown), which means that over 30% of the total particles are very likely to be trapped by the membranes.

Things are different when it comes to the transmission of the protein contaminants and the purity of sEVs. The transmission of the protein is defined as the ratio of total protein amount of the permeate to that of the feed. A significant stronger ability of removing protein contaminants was shown by both 300 and 500 kDa filter, with the transmission of protein being more than 60%, about three‐fold higher than that of 100 kDa membrane, indicating that the pore size of 100 kDa might be too small for most of the protein contaminants to pass through (Figure 2d). During the sEV separation process, a complex range of non‐vesicular materials might be co‐isolated. Therefore, a straightforward and quantitative method has been applied to estimate the purity of sEV samples by comparing the ratio of nano‐vesicle counts to protein concentration. Although both 300 and 500 kDa membranes were not as excellent as 100 kDa membrane in particle recovery, they showed a much better behaviour on the purification of sEV preparations due to their exceptional ability of removing protein contaminants. Compared to the performance of 100 kDa filter, a two to three‐fold higher purity was demonstrated by both 300 and 500 kDa membrane, showing that membranes with these two pore sizes might be promising candidates for sEV separation and purification while 100 kDa MWCO might have a wider use in final step sEV concentration instead of separation (Figure 2e).

In addition to the properties of the sEV preparations after filtration, the productivity of the membrane filtration process is also an important parameter that should be taken into consideration. In this case, the productivity of the membrane filtration process, expressed as particles produced per unit area of membrane per unit time, would largely depend on the permeate flux along with the sEV recovery, where the permeate flux is the quantity of the permeate flowing through the membrane per unit time and membrane area at a given pressure. As expected, under the same pressure, the shortest time was required for the 500 kDa membrane to process the 20 mL feed to 2 mL retentate, while the 300 kDa filter required more than twice the time, and even much longer for the 100 kDa filter (Figure 2f). Based on the volumetric flow with the time course, the permeate flux of every 30 s was calculated and shown in Figure 2g, where the permeate flux of all three membrane filters was constantly dropped, which might be due to the concentration polarisation or fouling. The permeate flux of the 500 kDa membrane was the highest in the first 90 s, after which no significant difference was found between the permeate flux values of 300 and 500 kDa (Figure 2g). The average productivity of each filter was calculated based on the final particle recovery and the time of the filtration process. Amongst the three, 500 kDa membranes showed the best productivity, which was two‐fold and six‐fold higher than that of the 300 and 100 kDa membranes, respectively (Figure 2h). Based on the above findings, for efficient separation of sEVs, a larger pore size, that is, 500 kDa MWCO in this case, is preferable.

### 3.2 Effects of stirring speed

The system hydrodynamics could have a distinct role to play in the membrane filtration process. Therefore, the rotary stirring speed was applied to generate different shear rates in the filtration system for the investigation of the shear effects on sEV separation. As the shear rate depends on both the geometry of the system and the stirring speed, the average shear rates at different stirring conditions were calculated from the computational fluid dynamics (CFD) simulation (see Supplementary Information) and shown in Table 1.

**TABLE 1:** Average shear rate at different stirring speeds.

Stirring speed (rpm)	Shear rate (s^−1^)
100	43.94
150	67.65
200	87.76
300	139.69
400	164.84

**FIGURE 3:**
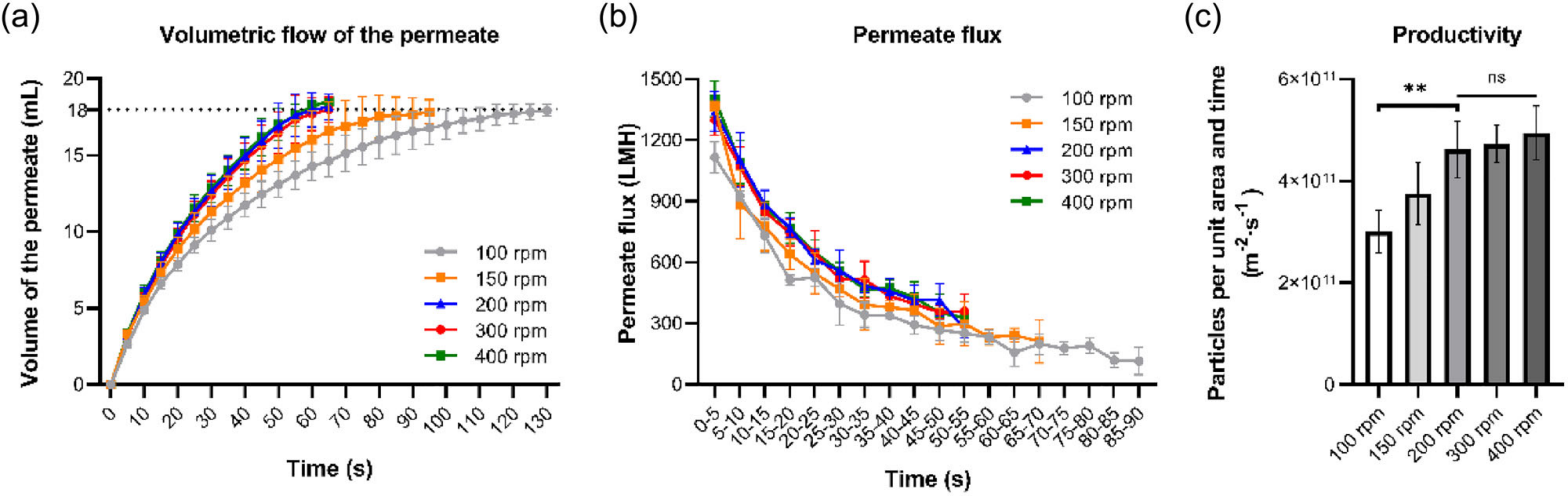
The effects of stirring speed on the sEV isolation process, using 500 kDa MWCO membrane under 10 psi applied pressure. (a) Volumetric flow rate of the permeate during the ultrafiltration under different stirring speeds. (b) Permeate flux during the ultrafiltration under different stirring speeds. (c) Average productivity of sEVs under different stirring speeds. MWCO, molecular weight cut‐off; sEVs, small extracellular vesicles.

No significant difference was found between the stirring speeds in the range of 100–400 rpm in terms of size distribution, recovery of nanoparticles, transmission of protein contaminants, and the purity of sEV preparations post‐filtration (Figure S2). However, as expected, it was found that the processing time required for the 20 mL feed to 2 mL retentate was continuously decreased and in turn, the permeate flux rose with the increase of stirring speed in the range of 100–200 rpm, although no significant difference was shown between the groups from 200 to 400 rpm (Figure 3a&b). As a result, with the increase in stirring speed, the average particle productivity was observed to first increase and then reach a plateau at 200 rpm (Figure 3c), given that the final recovery of particles was quite similar amongst the five groups. Therefore, since no significant difference was found in terms of the particle recovery and sEV purity under different stirring speeds, a stirring speed of 200 rpm seems to be a moderate setting to achieve the best sEV productivity and meanwhile minimise the shear effects so as that avoid the mechanical damage to the sEVs as much as possible.

### 3.3 Effects of operational pressure

**FIGURE 4:**
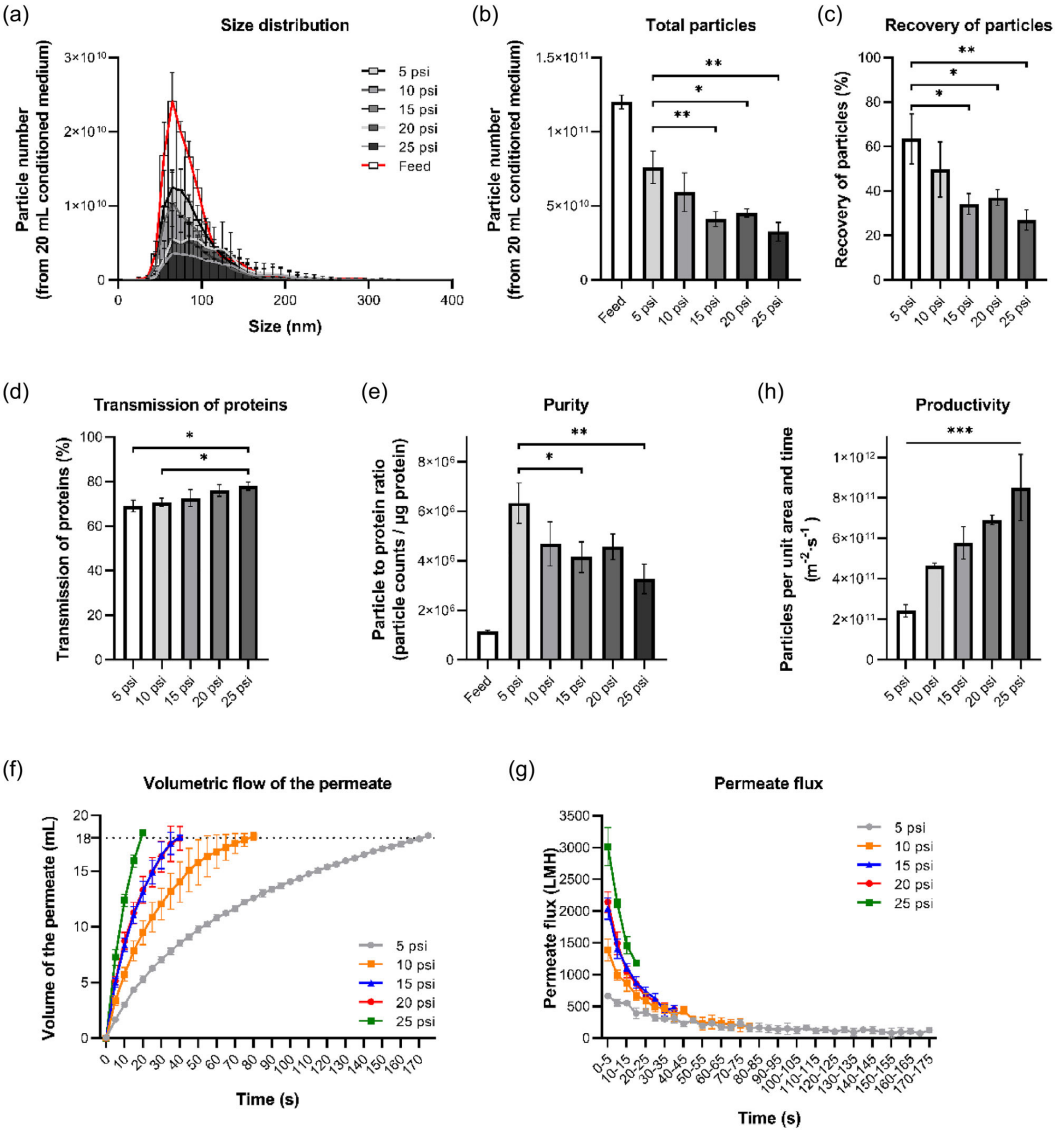
The effects of applied pressure on sEV isolation, using 500 kDa MWCO membrane under 200 rpm stirring speed. (a) Particle size distributions of the feed solution (CM) and the sEV preparations isolated under different pressures. (b) Total particle numbers of CM and the sEV preparations. (c) Recovery of particles from CM under different pressures. (d) Transmission of contaminated proteins during the ultrafiltration under different pressures (e) Purity (particle to protein ratio) of CM and the sEV preparations. (f) Volumetric flow rate of the permeate during the ultrafiltration under different pressures. (g) Permeate flux during the ultrafiltration under different pressures. (h) Average productivity of sEVs under different pressures. CM, conditioned medium; MWCO, molecular weight cut‐off; sEVs, small extracellular vesicles.

Pressure is the major driving force of an ultrafiltration process. Different transmembrane pressure (TMP) created by the compressed air was tested to optimize the ultrafiltration‐based separation protocol of sEVs. Figure 4a demonstrated that the size distribution of the sEV preparations obtained under different pressures was consistent with typical sEV profiles. In the range of 5–25 psi, the higher the pressure applied, the less the particles and proteins can be retained, which means a worse particle recovery (Figure 4b&c) but better behaviour in removing protein contaminants (Figure 4d) with the increased pressure. Compared to the transmission of protein contaminations, it seems that the particle recovery might be more sensitive to the pressure change as it dropped more than half (from around 63% to 27%) while the removal of the proteins only increased by 9% (from 69% to 78%) when the pressure increased from 5 to 25 psi. As a result, the purity of the sEV preparations also dropped when higher pressure was applied (Figure 4e). Again, the particle amount in the permeate occupied less than 4% of the total particles (data not shown), with no significant difference between that of different pressures, suggesting more particles might be trapped by membranes with higher pressure applied. Nevertheless, as expected, the permeate flux increased with the pressure (Figure 4f&g), which in turn led to significant growth of sEV productivity (Figure 4h). In view of the above findings, 5 psi was chosen as the most appropriate pressure out of a reason that it demonstrated both the best recovery and purity of sEV products, which should not be compromised although higher pressure would give a better productivity of sEVs.

### 3.4 Effects of feed concentration (direct dilution) and diafiltration

Introducing a washing buffer or fresh solvent to the ultrafiltration system can be a promising solution to further remove or lower the concentration of those contaminants and facilitate the separation to obtain purer sEV preparations. Diafiltration is a commonly used method to wash out the contaminant from the solution, which consists of repeated dilution and concentration steps to achieve buffer exchange. In this case, 20 mL original feed was first concentrated to 10 mL by 500 kDa MWCO membrane, followed by adding an equal amount (10 mL) of PBS and then diafiltrated to 10 mL again, which is recorded as one DV and was repeated for several times. After the final time of diafiltration, the 10 mL retentate was concentrated to 2 mL as the final retentate by the same membrane. The groups that conducted 2 times (2 × 10 mL PBS in total) and 6 times (6 × 10 mL PBS in total) diafiltration were termed group DV2 and group DV6, respectively. A direct dilution process with the addition of the same PBS volume was conducted parallelly for comparison, that is, 20 mL original feed was diluted two‐fold with 20 mL PBS (termed as group 0.5×) or 4‐fold with 60 mL PBS (termed as group 0.25×) before the filtration by 500 kDa MWCO membrane. A control group termed group 1× (DV0) was also performed, in which 20 mL original feed was directly filtered by a 500 kDa MWCO membrane to 2 mL retentate, without adding any PBS.

**FIGURE 5:**
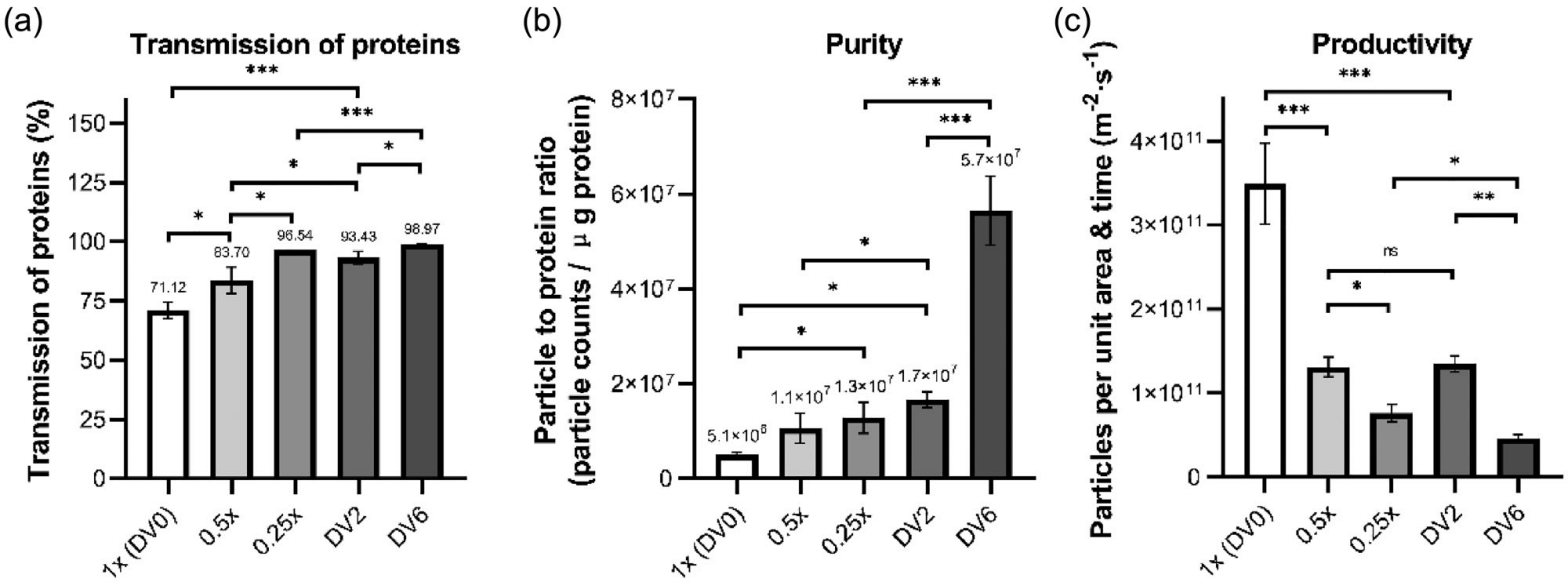
The effects of direct dilution and diafiltration on sEV isolation, using 500 kDa MWCO membrane under 200 rpm stirring speed and 10 psi applied pressure. (a) Transmission of contaminated proteins during the ultrafiltration. (b) Purity (particle to protein ratio) of the sEV preparations. (c) Average productivity of sEVs. Both dilution and diafiltration increased protein transmission, leading to purer sEV preparations, while reduced productivity, with higher dilution folds or DVs applied. The DV6 group removed nearly 99% of protein contaminants and significantly boosted purity—about 10 times greater than the control group 1× (DV0). With an equal volume of the fresh buffer added, the DV6 group achieved much higher purity compared to the four‐fold dilution group (0.25×) with only a slight reduction in productivity. DV, diafiltration volume; MWCO, molecular weight cut‐off; sEVs, small extracellular vesicles.

The performance of nanoparticle recovery was quite similar amongst all the groups (Figure S3a, b), indicating that the dilution and diafiltration process will not lead to further loss of the particle. Both the dilution groups and the diafiltration groups demonstrated an increase in the protein transmission (Figure 5a) and thus purer sEV preparations (Figure 5b), with higher dilution folds or more DV applied. Particularly, six diafiltration volume (DV6) group removed almost 99% of the protein contaminants and demonstrated a remarkable increase in sEV purity, for about 10‐fold higher than that of the control group. In addition, with the same volume of the buffer added, the behaviour of the diafiltration process was constantly superior to that of the direct dilution process in both removal of protein contaminants and sEV purity. Compared to the 4‐fold dilution group (0.25×), the DV6 group showed a four to five‐fold higher purity. Nevertheless, as expected, although the permeate flux was increased when the feed was more diluted, the processing time was extended (Figure S3c–e) and the productivity was dropped (Figure 5c) for the groups with higher dilution folds or more diafiltration repeats, out of the reason that the total processing volume increased, that is, 40 mL for the groups of 0.5× and DV2, 80 mL for the groups of 0.25× and DV6. However, the groups of two‐fold dilution and two DV gave a comparable productivity, and the productivity of DV6 group was only slightly lower than that of 0.25× group (Figure 5c). Considering that diafiltration process played an excellent role in sEV purification, it should be adopted to the current membrane‐based sEV separation process. To obtain high quality sEV preparations with required purity, the number of DV need to be determined. According to above results, DV of six is recommended for sEV separation and a safety factor of one more DV could be added to make the final removal rate over 99%.

### 3.5 Development of sEV purification process: Fed‐batch ultrafiltration combined with diafiltration (UF/DF)

**FIGURE 6:**
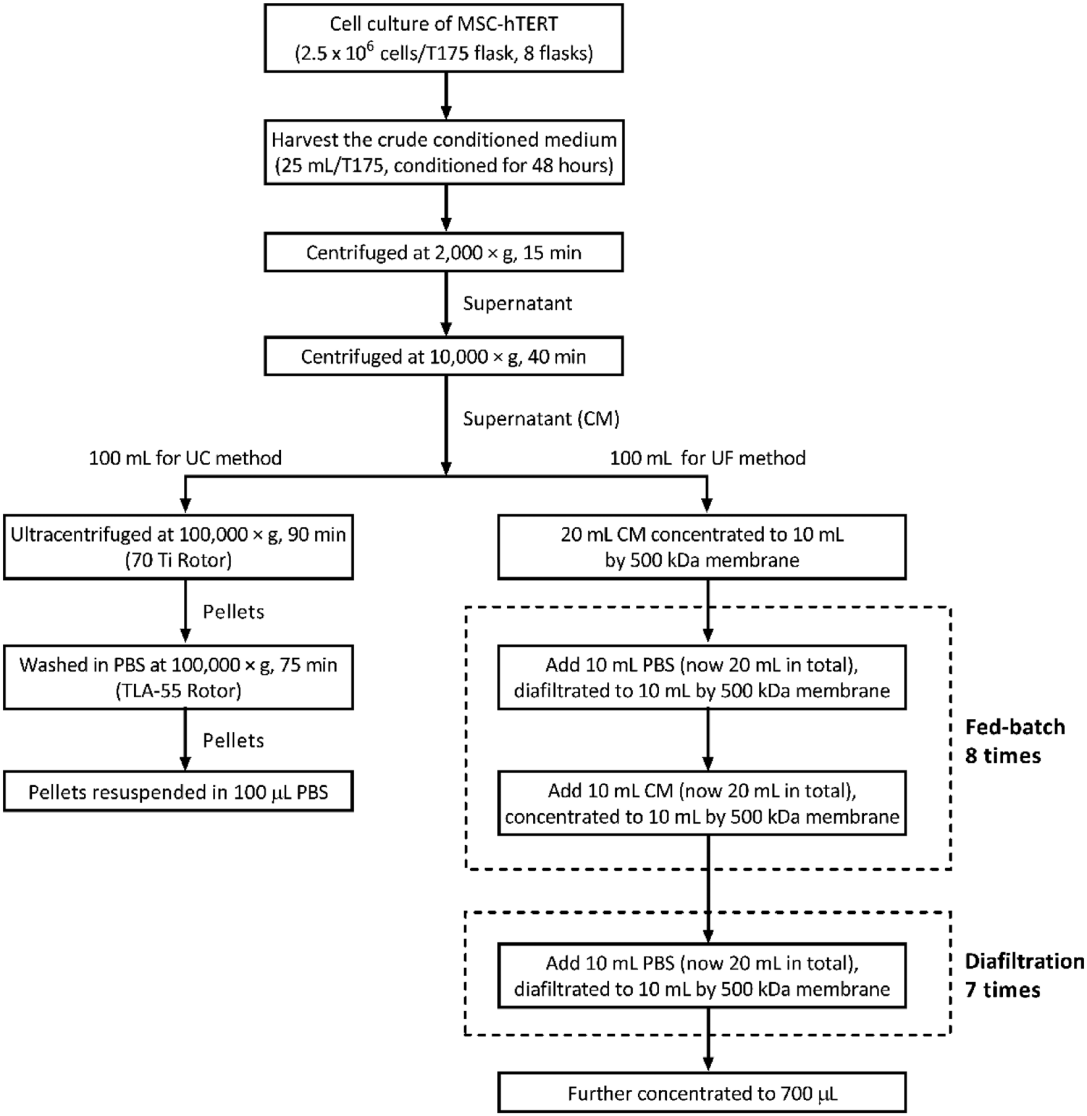
Flow chart of the fed‐batch UF/DF process and UC process. UC, ultracentrifugation; UF/DF, ultra‐diafiltration.

Based on the previous results, the optimal parameters were adopted to our membrane ultrafiltration protocol for sEV separation, that is, the conditioned medium would go through a pre‐concentration step to half of the volume, 7 diafiltration steps, and a final concentration step, using a 500 kDa MWCO membrane under a condition of 200 rpm stirring speed and 5 psi applied pressure. However, as shown in Figure 2g, with a starting volume of 20 mL 1× conditioned medium, the permeate flux dramatically declined during the process due to concentration polarisation and potential membrane fouling and thus led to inefficiency at a later stage of ultrafiltration, which reflected a limited processing capacity of a single membrane disc. To enlarge the processing capacity of a single membrane disc and meanwhile maintain the permeate flux and the productivity at an acceptable level, here we developed a fed‐batch UF/DF protocol as shown in Figure 6. A total of 100 mL conditioned medium was divided and fed to the system in batches, and a concentration and diafiltration step was added between each feed.

**FIGURE 7:**
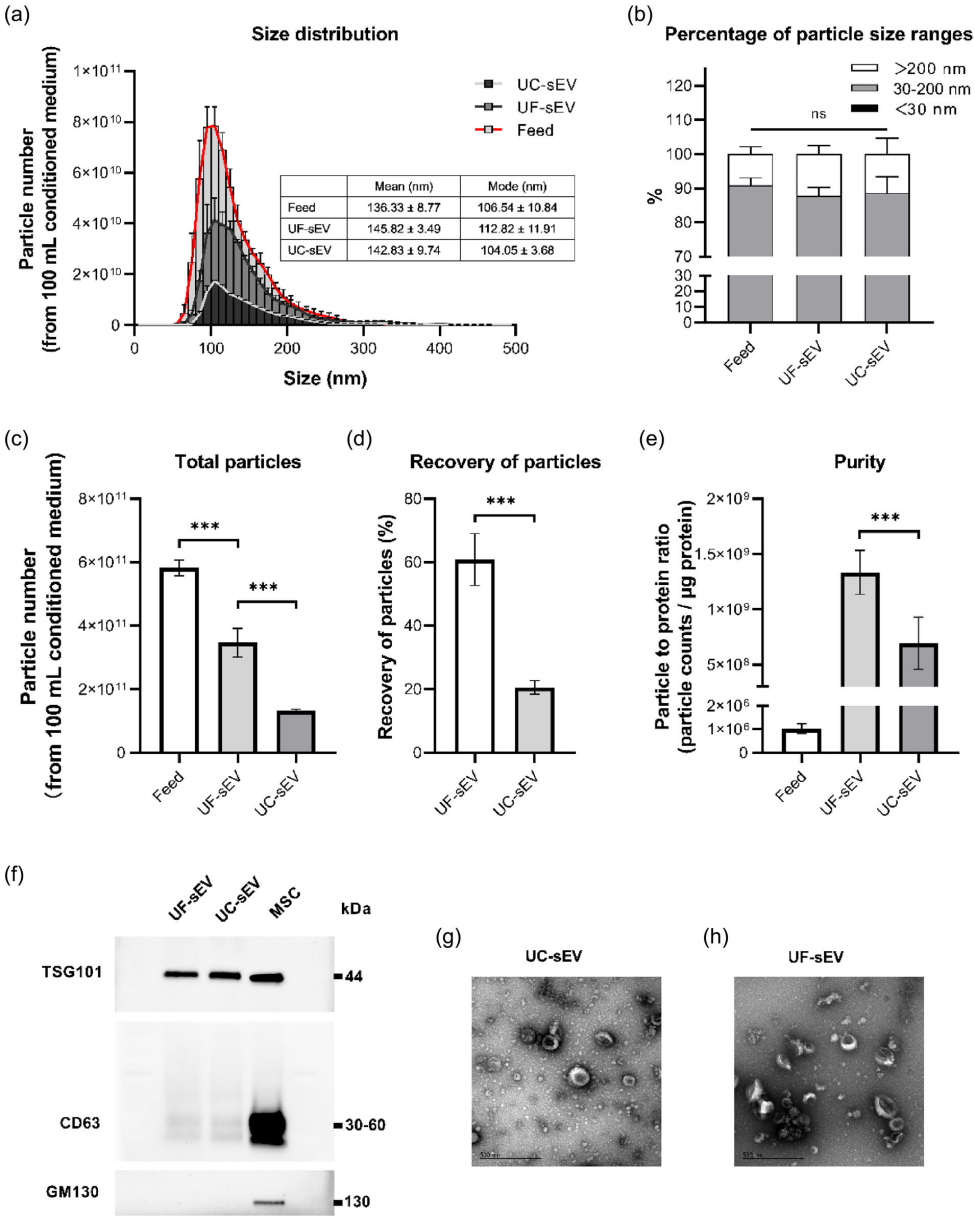
Characterisation of sEV preparations from fed‐batch UF/DF (UF‐sEV) and UC (UC‐sEV). (a) Particle size distributions of UF‐sEV, UC‐sEV and feed solution (CM). (b) Percentage of particles of different size ranges. (c) Total particle numbers of UF‐sEV, UC‐sEV and CM. (d) Recovery of particles from CM by fed‐batch UF/DF and UC. (e) Purity (particle to protein ratio) of UF‐sEV, UC‐sEV and CM. (f) Western blot results of UF‐sEV, UC‐sEV and their parent cells. (g) TEM image of UC‐sEV (scale bar represents the length of 500 nm). (h) TEM image of UF‐sEV (scale bar represents the length of 500 nm). CM, conditioned medium; sEVs, small extracellular vesicles; UC, ultracentrifugation; UF/DF, ultra‐diafiltration.

The separation result of this fed‐batch UF/DF method were compared to that of the UC method, the most used sEV isolation method so far. Compared to UC, the fed‐batch UF/DF reduced the processing time of sEV isolation. It took about 210 min for the fed‐batch UF/DF method including about 1 h time consumed for manually configuring and taking apart the ultrafiltration system, which can be further shortened to less than 2.5 h after automation, while UC‐based method needs at least 4 h (Table 2). In addition, fed‐batch UF/DF method showed a remarkable ability in sEV recovery (Figure 7c, d), with about 60.88% particles being recovered from the conditioned medium, which was almost three‐fold higher than that of the UC method (20.59%). Moreover, although more PBS buffer was required during the UF‐based isolation process (Table 2), the purity of sEV preparations (particle to protein ratio) from the fed‐batch UF/DF method was about 1300‐fold higher than that of the feed solution, and was increased from 6.93 × 10^8^ to 1.33 × 10^9^ (nearly doubled), compared to that of the UC method (Figure 7e). The size distribution of sEV preparations isolated by both methods was consistent and corresponding to the typical sEV profiles (Figure 7a), with about 90% of the particles ranging from 30 to 200 nm (Figure 7b). sEV preparations from both methods were positive for typical EV markers such as CD63 and TSG101, and negative for intracellular compartment markers, like GM130, which confirmed the purity of the final sEV samples (Figure 7f). Classic cup‐shaped EV particles were found in TEM images from both methods, and a higher sEV purity from the fed‐batch UF/DF method was further confirmed as less contaminants were found in the background of the image (Figure 7g, h).

**TABLE 2:** Comparison of fed‐batch UF/DF method and UC method for sEV isolation.

	Fed‐batch UF/DF	UC
Processed conditioned medium (mL)	100	100
PBS required (mL)	150	≤25
Obtained sEV preparation (µL)	700	100
Processing time (min)	210	≥240
Recovery of particles (%)	60.89 ± 8.16	20.59 ± 2.15
Purity (particle counts/µg protein)	1.33 × 10^9^ ± 1.99 × 10^8^	6.93 × 10^8^ ± 2.35 × 10^8^

Abbreviations: sEVs, small extracellular vesicles; UC, ultracentrifugation; UF/DF, ultra‐diafiltration.

## 4 DISCUSSION

### 4.1 Separation performance with different ultrafiltration design and operational parameters

Membrane filtration could be a promising and industrial applicable sEVs isolation method for downstream studies and clinical applications. In this study, we investigated the effects of the different filtration parameters on sEV recovery, purity as well as productivity, then further developed an ultrafiltration‐based protocol combined with fed‐batch and diafiltration process for sEV separation, which has been approved to achieve a much more excellent recovery of sEVs with higher purity and less processing time, compared with UC, the most commonly used method for sEV isolation in the lab.

The ultimate goal for this separation is to achieve high recovery and excellent purity of the desired sEVs. This needs to retain the sEVs in the desired size range as much as possible and to remove smaller free proteins and other contaminants. A suitable membrane, that provides a barrier to retain sEVs and to transmit protein, would be the first to consider.

It seems that 500 kDa MWCO is the membrane pore size that allows the highest productivity without compromising the purity of sEVs amongst the three. MWCO is defined as the molecular weight that 90% of the molecules cannot pass through the membrane. The pore size of 100, 300, and 500 kDa MWCO membrane can be estimated by Equation (1), and the estimated diameters are 6.13, 8.84, and 10.48 nm, respectively (Erickson, 2009).

(1) $$R_{\min}=0.066M^{1/3}$$

where $R_{\min}$ is the radius in the nanometer, and M is the molecular weight in Dalton.

According to the technical guidance from companies like Pall Corporation and Sterlitech, as a rule of thumb, a MWCO in the range of 3–6 times smaller than the molecular weight of the targets is recommended, with an optimum flow rate using three times smaller MWCO (SterliTECH Tip: What is Molecular Weight Cut‐Off (MWCO)?, Pall Laboratory, 2022). As a result, knowing that the size of the sEVs is 30–200 nm, 500 kDa with a pore size of around 10 nm in diameter could be a suitable choice for sEV separation, which also maximises the transmission of protein contaminants at the same time and thus improves the purity of sEV preparations.

It is noted that the large membrane pores, like 500 kDa MWCO, may have a higher tendency of pore blocking, compared to membranes with smaller pores, which explains the sharp flux decline observed in Figure 2g. Concentration polarisation is a common phenomenon during membrane filtration, which is caused by the reversible accumulation of components close to the surface of the membrane. It can lead to severe fouling problems of the membrane, including complete/partial/internal pore blocking and filter cake (Giacobbo et al., 2018). Both concentration polarisation and fouling can cause higher resistance for transport as well as flux decline during the pressure‐driven filtration, and will further affect the lifetime of the membrane (Crozes et al., 1997; Wetterau et al., 1996). The tendency of flux decline can be closely related to the interaction between the components in the feed and the membrane pores (Kang & Choo, 2003). Particles might be more easily to be immobilized or trapped by larger membrane pores like 300 and 500 kDa MWCO, while tend to roll off the membrane surface with lower MWCO under the same operational condition (Choi et al., 2005; Md Yunos et al., 2019). This problem could be alleviated by changing the hydrodynamic conditions, surface modification of the membrane, and regular cleaning, which should be considered in the process development in the future (Field et al., 1995).

The performance of membrane ultrafiltration, including diafiltration, for bioseparation is affected by operational (stirring speed, TMP, and temperature) and physiochemical parameters (concentration, solution pH and ionic strength). The former affects concentration polarisation and possible fouling onto the membrane. The latter affects the electrostatic interactions between sEVs, proteins and the membrane surface, and amongst themselves.

Higher permeate flux and greater productivity can be achieved by the increase of the stirring speed (shear rate) which reduces concentration polarisation (Jönsson, 1993; Lee et al., 2005; Md Yunos et al., 2019; Quezada et al., 2021). However, the permeate flux was not that sensitive to the change of stirring speed over 200 rpm. This is because, at a higher shear rate, the concentration polarisation is minimised and no longer a limiting factor (Kang & Choo, 2003; Md Yunos et al., 2019). Further study of cross‐flow filtration for sEV separation is recommended, which could potentially provide an improved hydrodynamic condition for protocol optimisation.

Lower operational pressure is recommended for sEV separation for higher particle recovery and thus excellent purity of sEVs. This might be because more severe fouling (pore blocking) can be caused with higher pressure applied, as more particles might be pushed to the membrane and into the membrane pores, which would intensify pore blocking and eventually lead to poor particle recovery (Md Yunos et al., 2019). The increased local concentration of sEVs near the membrane surface would reduce the electrostatic interaction (repulsion) between the sEV particles and hence form a denser cake. It must also be pointed out that the high operational pressure and high initial permeate flux increase the local shears the sEV particles experience, which may lead to breakages of sEVs and hence unwanted product loss. This is also a factor to consider when choosing the membrane pore size. However, we did not explore pressures lower than 5 psi in this study due to experimental equipment constraints, as the minimum scale on the pressure regulator assembly used was 5 psi. Therefore, it is unclear whether a pressure lower than 5 psi would significantly enhance purity or particle recovery but further reduce productivity. As the key challenge is to find a balance between maintaining adequate productivity and achieving higher purity and yield, future work could be carried on with lower pressures to better optimize this balance.

The concentration of the feed solution (conditioned medium) also affects the retention and transmission of sEVs and protein contaminants. Generally, a lower concentration would allow smaller proteins to pass the membrane pores, resulting in a higher purity of the retained sEVs. Dilution of the feed solution might help with sEV separation. This result may be explained by the fact that the decrease of the feed concentration might ease the problem of concentration polarisation and fouling, which favours the throughput of the ultrafiltration system and the transmission of protein contaminants to some extent, and thus increases sEV purity. It might also be explained that a higher concentration of the feed could lead to an increase in viscosity and therefore hinder the efficiency of the separation (Jones & Talley, 1933).

Diafiltration process shows an exceptional performance in removal of protein contaminants during the sEV separation process. Diafiltration usually involves pre‐concentration of feed solution, dilution of feed liquid by adding fresh washing buffer to replace the volume of the lost permeate, and a volume reduction step by ultrafiltration to wash contaminants away from the retained particles (Hoffmann et al., 2018). In this case, concentration to half of the initial feed volume was performed not only to reduce the overall sample volume and the required amount of washing buffer for easier liquid handling and economic purpose, but also to avoid severe polarisation problem which might be caused by further concentration of the feed. In contrast to direct dilution of the feed, with the same volume of the buffer added, the diafiltration process demonstrated an even more remarkable ability in sEV purification. This might be explained by the sequential dilution and buffer exchange process of the diafiltration. The permeable components are sequentially diluted, and half of the feed volume is continuously replaced by the fresh buffer during each diafiltration step, together with the volume reduction during the pre‐ and final‐concentration step, leading to 99.8% (see Supplementary Information) of the original feed volume been replaced by PBS washing buffer after DV6. Compared with the value of 97.5% (see Supplementary Information) of the direct dilution 0.25× group, a larger portion of the original solution was removed or replaced by diafiltration in DV6 group and thus enhanced the removal of contaminants and improved the purity of sEVs post‐separation, although with the addition of the same volume of PBS washing buffer. The same reason can also be applied to the DV2 and 0.5× groups.

### 4.2 Process development

The ultrafiltration design and parameters of the sEV separation protocol were optimised based on the previous results, that is, the conditioned medium would be pre‐concentrated to half of the volume, diafiltered with PBS for seven times and then concentrated again with a 500 kDa MWCO membrane at 200 rpm stirring speed and 5 psi. The reason for concentrating the 20 mL conditioned medium to 10 mL and diluting it 1:1 with PBS, rather than concentrating it further to 2 mL and diluting with a larger volume of PBS, was to minimize significant changes in sample concentration during the process. Concentrating to 2 mL would result in a sharp increase in solute concentration, leading to concentration polarisation and membrane fouling during filtration as well as reducing the permeate flux and the system's efficiency and potentially leading to the retention of impurities. While concentrating to 2 mL might seem like it would allow for a larger volume of buffer to be added and a greater dilution factor, it actually slows the process. This is because the time to concentrate further would increase, and any potential fouling would further slow down or disrupt the diafiltration process. The 10 mL concentration step offers a practical balance, maintaining the process efficiency by keeping a relatively high buffer exchange rate without introducing significant delays or disruption that can arise from overly concentrated samples. In addition, in order to maintain consistency with the volume used in the UC method for comparison, 100 mL of media was applied to the ultrafiltration experiments, which was five times greater than the initial experimental design of 20 mL. With the limitation of a fixed membrane area in the HP4750 Stirred Cell System, fed‐batch concentration is adopted to our sEV separation protocol before the diafiltration separation step. Compared to one‐time direct feed concentration to half volume (e.g., from 100 to 50 mL), greater volume reduction and thus easier liquid handling as well as higher processing capacity can be achieved by fed‐batch concentration process, that is, 100 mL feed solution was divided into several fractions, fed to the system in batches and concentrated to half volume sequentially, making the final volume of the retentate to 10 mL at the last fed‐batch (Figure 6). However, higher concentrations in the retentate can be caused as the feed solution is continuously added and concentrated, which would also lead to serious concentration polarisation and fouling problems, and thus poor throughput of the membrane. Therefore, a diafiltration step was designed between each batch of feed concentration to preliminarily dilute and wash away part of the contaminants in order to maintain an acceptable permeate flux and increase the processing capacity of the membrane (Lutz, 2015). The downstream diafiltration process can be operated once all the feed solution has been accommodated and concentrated.

It is important to reserve the characteristics of the sEVs, no matter what separation method is used. Here, we isolated sEVs from the fed‐batch UF/DF method and compared them with those from the conventional UC method. In this study, sEVs with characteristic size distributions, classic morphology and typical expression of EV markers were successfully isolated by our fed‐batch UF/DF method, showing comparable characteristics to those from the UC method. In addition, this method also exhibited its superior ability in sEV separation, in contrast to the UC method.

Co‐isolation of non‐vesicular contaminants is the major problem that needs to be addressed, which may influence subsequent studies including biomarker exploration, functional studies, clinical trials as well as further applications (Abramowicz et al., 2018; Takov et al., 2019; Webber & Clayton, 2013), therefore, the purity of the sEV preparation is of critical importance (Théry et al., 2019). Although the purity of the sEV preparations from both fed‐batch UF/DF and UC were confirmed by the western blot results, the further difference between the two was unable to be identified due to the lack of sensitivity. A method based on measuring the particle‐to‐protein ratio was proposed by Webber et al., allowing quantitative comparison of purity between different sEV preparations (Webber & Clayton, 2013). By this means, a stronger capability of sEV purification was shown by our self‐developed fed‐batch UF/DF method, with a significantly higher particle‐to‐protein ratio than that of the traditional UC method, which was also supported by the TEM images in Figure 7. However, it should be noted that although this method provides a straightforward comparison of the purity between different sEV preparations, further quantitative biochemical studies about various specific non‐EV contaminants should be conducted in the future to provide a deeper insight into the co‐isolation problems of different sEV separation methods.

The recovery of sEVs is another crucial factor in evaluating an sEV separation method. A three‐fold particle recovery was accomplished by the fed‐batch UF/DF method compared with UC. This result may be due to that the high shear forces caused by UC destroy particle integrity and cause particle aggregation, leading to a sharp drop in the total particle number, which was also reported by Cvjetkovic et al., 2014; Linares et al., 2015. In contrast, our fed‐batch UF/DF method was designed to operate under a relatively gentle condition with low pressure and stirring speed for the purpose of minimising the mechanical damage to the sEV particles. Haraszti et al. and Heinemann et al. also reported similar findings that higher quantities of sEVs were produced by filtration than UC method, although the tangential flow filtration method and the sequential filtration method they applied was slightly different from our fed‐batch UF/DF (Nakai et al., 2016; Ng et al., 2019). It is also essential to bear in mind that an assumption was made that all the particles detected by the NTA method were counted as vesicles due to the limitation of the detection technique, which might not be strictly true.

An ideal sEV separation method should also be time‐efficient, user‐friendly, cost‐effective, and easy to scale up. For a processing volume of 100 mL, it seems our fed‐batch UF/DF method in the manual operation mode does not show straightforward advantage in saving processing time and simplifying the separation step. However, considering that the yield of fed‐batch UF/DF method was about three folds higher than that of the UC, as a result, three folds of consumable costs and processing times were required by UC to obtain an equal amount of sEVs, that is 12 h for UC versus 3.5 h for fed‐batch UF/DF. In addition, the processing time of the UC method mainly depends on the processing capacity of the ultracentrifuge, and was impossible to shorten for each batch, while automation of our fed‐batch UF/DF protocol is highly possible to be accomplished to shorten the processing time to less than 2.5 h (almost half of the UC method), which in turn would further decrease the processing time and largely reduce manual intervention, and therefore decrease the labour cost. Moreover, the capital costs of an ultracentrifuge and rotors for UC method are extremely higher than that of filtration devices for ultrafiltration, which means our ultrafiltration‐based method would be more affordable and accessible.

While this study demonstrates a scalable and efficient method for isolating sEVs using ultrafiltration, a limitation of this study is that it does not investigate the potential effects of mechanical forces generated during the process on the biological integrity of the sEVs. Mechanical stress on sEVs during ultrafiltration may induce damage to the sEVs, which could impact their biological properties, interaction with target cells, and functionality in relation to their therapeutic applications. Future studies are needed to assess the potential mechanical damage to sEVs and their implications for their biological activity, as well as optimise the process to preserve sEV integrity for downstream therapeutic use.

## 5 CONCLUSIONS

To summarise, the main findings in this study are shown below: 500 kDa MWCO might be the most suitable membrane pore size for efficient purification and production of sEVs. Higher sEV productivity can also be achieved by increasing stirring speed to a certain level, and lower operational pressure allows a gentle separation of sEVs for excellent particle recovery and sEV purity. By introducing several diafiltration steps, the purity of the sEV preparations can be further improved, with about 99% of the protein contaminants being removed after six times of isovolumetric diafiltration. The fed‐batch UF/DF method developed based on these findings was able to achieve a three‐fold higher yield and nearly doubled purity of sEVs compared to the traditional UC method, which could be a very promising sEV separation method for downstream studies with extra advantages of efficient production and low costs. Due to its scalable and automatable properties, it also has great potential in industrial‐level sEV manufacture for clinical applications. Nevertheless, a limitation of this study was that only the biophysical properties of sEV preparations were investigated while the biological and functional integrity of the sEV samples post‐separation was unclear. Therefore, the biological properties and the functional capacity of sEVs isolated by both methods should be evaluated and compared. In addition, the fed‐batch UF/DF protocol can be further developed in the future. For example, tangential flow filtration should be considered in order to provide better hydrodynamic conditions, which may largely alleviate the problem with concentration polarisation and improve sEV productivity. Also, it might be a good idea to test the (continuous) constant volume diafiltration (CVD) mode, where the fresh buffer is continuously introduced to the filtration system at the same rate of withdrawing the permeate, leading to a more efficient and gentle buffer exchange compared to the discontinuous diafiltration. A pilot scale sEV isolation system with an automatic separation program could then be developed for the manufacture of high‐end MSC‐sEV products.

## AUTHOR CONTRIBUTIONS

**Rui Lei**: Data curation (lead); investigation (lead); methodology (lead); writing—original draft (lead); writing—review and editing. **Shuai Ren**: Writing—original draft (supporting). **Hua Ye**: Funding acquisition (lead); supervision (equal); writing—review and editing (equal). **Zhanfeng Cui**: Conceptualization (lead); funding acquisition (supporting); supervision (equal); writing—review and editing (equal).

## CONFLICT OF INTEREST STATEMENT

The authors declare that they have no competing interests.

## Supporting information



Supporting Information

## Data Availability

Data sets generated during this study are available from the corresponding authors upon reasonable request.
