# Supplementary material for: Purification of mesenchymal stromal cell‐derived small extracellular vesicles using ultrafiltration
Source: J Extracell Biol. 2025 Jan 17;4(1):e70030. doi: 10.1002/jex2.70030 (PMC11739894; doi:10.1002/jex2.70030)
Supplement: Supplementary file 1 — Supporting Information [file JEX2-4-e70030-s001.docx]

## Supplementary Information

### Computational Fluid Dynamics (CFD) Simulation

A computational fluid dynamics (CFD) simulation was conducted in the commercial software ANSYS FLUENT (Version 2020 R2) to explain the fluid environment during the ultrafiltration, especially for the fluid shear rate and velocity magnitude in the stirred cell device under different rotary stirring speeds. The whole filtering process was illustrated in Supplementary Figure 1a. Under a constant air pressure (10 psi), 20 mL sample solution was mixed by the adjustable rotated impeller and filtered by the chosen membrane (500 kDa MWCO), which was mounted at the bottom of the stirred cell. The simulation of this part only predicts the impeller's influence under varying rotary stirring speeds on the sample. The filtering process and efficiency were not considered.

Supplementary Figure 1b&c indicate the engineering drawing of the working space and the impeller, which reflect the critical geometry parameters. The sample solution filled in a reversed circular truncated cone, which has a 43mm top diameter (D_up_), 20mm height (H_SC_) and 32mm bottom diameter (D_down_). The rotated impeller has the below-listed feature: 22mm for D_impeller_, 15mm for H_impeller_, 6mm for W_inner_ and 5.5mm for H_inner_. All the models were built by the Inventor Professional 2020 (Autodesk, ltd) and exported to STP files for transferring to ANSYS FLUENT. The models were then meshed by the Ansys Mesh to 1mm tetrahedral units. The whole meshed models contain 46900 nodes and 229618 elements. All the other settings were Ansys Mesh default.

This CFD analysis is considered as a transient processing due to the outflow from the stirred cell. The pressure-based type was chosen and 9.81 m/s^2^ gravitational acceleration was set. Two phases were set during the simulation: gas (air) and fluid. The property of the fluid was similar to water liquid (turbulent and incompressible) with a measured average viscosity of 1.337$\times$10^-3^ kg/(m$\cdot$s). The Eulerian multiphase model described the interaction between phases with the grace coefficient and 0.073 surface tension coefficient. The diameter of the air was set as 0.0015mm. The Standard K-epsilon model explained the turbulence. 10 bar pressure was exerted from the top of the filtration chamber. The Sliding Mesh approach was used to simulate the rotation of the central impeller at 100, 150, 200, 300, and 400 rpm.

At the post-simulation step, the fluid shear rate and velocity magnitude were plotted by the CFD-Post (ANSYS, ltd) in Supplementary Figure 1d-q. The velocity magnitude is the absolute value of velocity which refers to the speed or rate at which a liquid unit moves in a particular direction in this project. The vectors show the direction of liquid motion. The fluid shear rate measures the rate at which a fluid is sheared or deformed. Due to there being a 3D CFD simulation, the fluid shear rate was calculated by the Equation below:

$Shear Rate =\left[ 2\left( \left( \frac{{\partial U}_{x}}{\partial x} \right)^{2}+\left( \frac{{\partial U}_{y}}{\partial y} \right)^{2}+\left( \frac{{\partial U}_{z}}{\partial z} \right)^{2} \right)+\left( \frac{{\partial U}_{x}}{\partial y}+\frac{{\partial U}_{y}}{\partial x} \right)^{2}+\left( \frac{{\partial U}_{x}}{\partial z}+\frac{{\partial U}_{z}}{\partial x} \right)^{2}+\left( \frac{{\partial U}_{y}}{\partial z}+\frac{{\partial U}_{z}}{\partial y} \right)^{2} \right]^{\frac{1}{2}}$

where $U_{x}$, $U_{y}$, and $U_{z}$ are the velocity components in space.


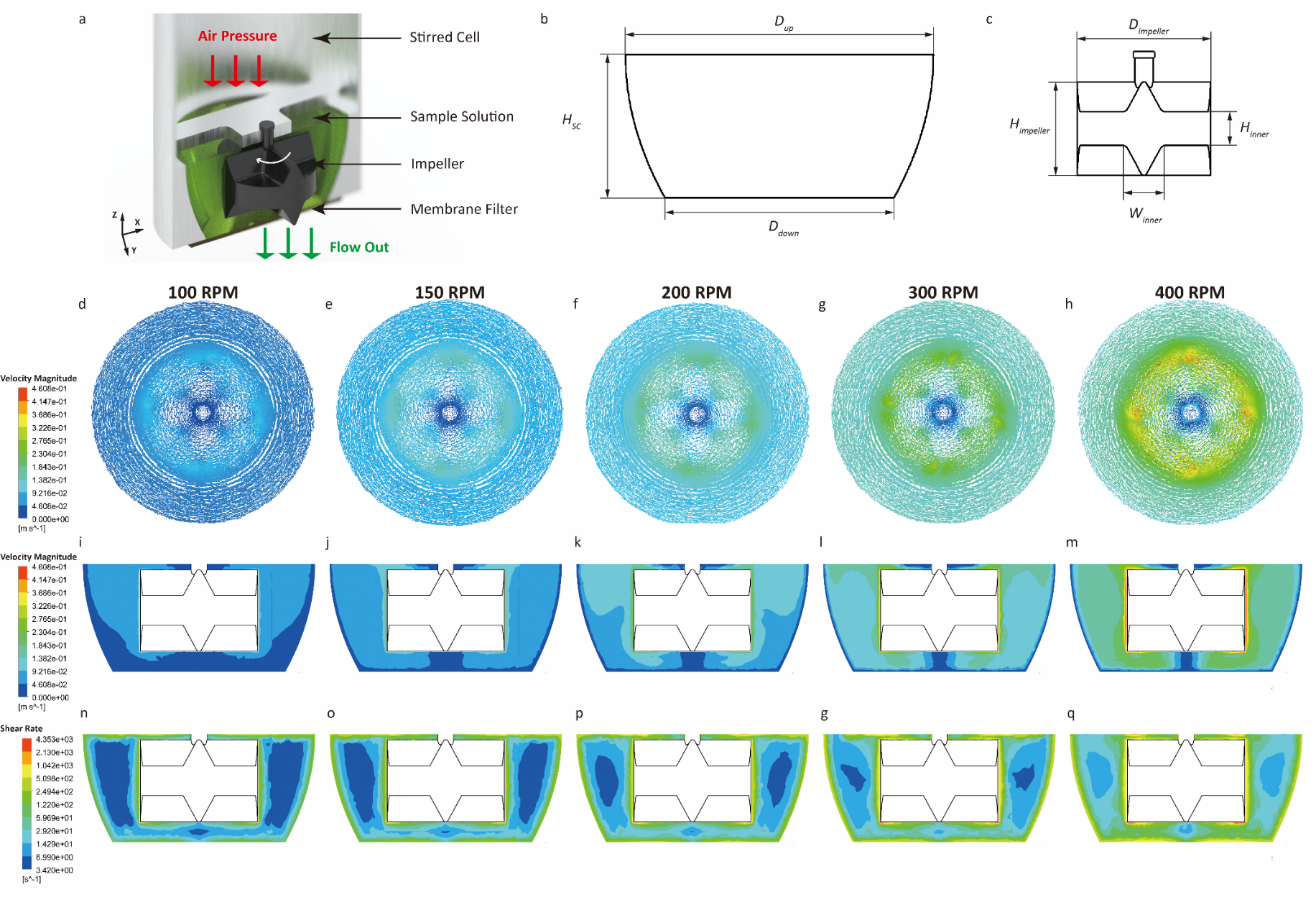


Supplementary Figure 1 Computational fluid dynamics simulation. (a) The schematic diagram of the stirred cell working area. (b) and (c) The engineering drawing of the working space and the impeller. Vector figure of the velocity magnitude (d-h) at the top view of the stirred cell under the stirring conditions of 100, 150, 200, 300, 400 rpm, respectively. Contour figure of the velocity magnitude (d-m) and shear rate (n-q) at the cross section of the stirred cell under the stirring conditions of 100, 150, 200, 300, 400 rpm, respectively.

### The Effects of Stirring Speed on the Properties of the sEV Preparations


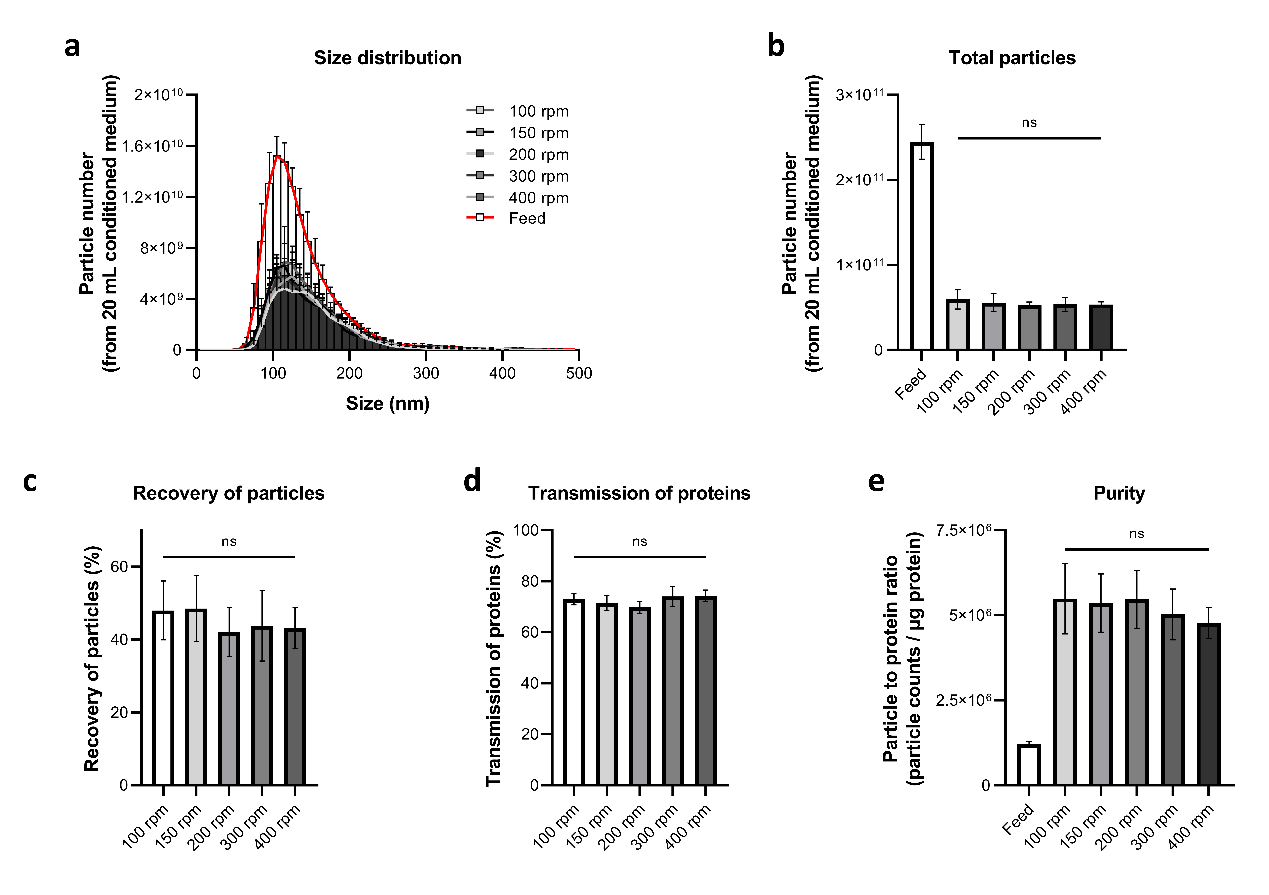


Supplementary Figure 2 The effects of stirring speed on the properties of the sEV preparations. (a) Particle size distributions of the feed solution (CM) and the sEV preparations isolated under different stirring speeds. (b) Total particle numbers of CM and the sEV preparations. (c) Recovery of particles from CM under different stirring speeds. (d) Transmission of contaminated proteins during the ultrafiltration under different stirring speeds. (e) Purity (particle to protein ratio) of CM and the sEV preparations.

### The Effects of Direct Dilution and Diafiltration on Particle Recovery and Isolation Process


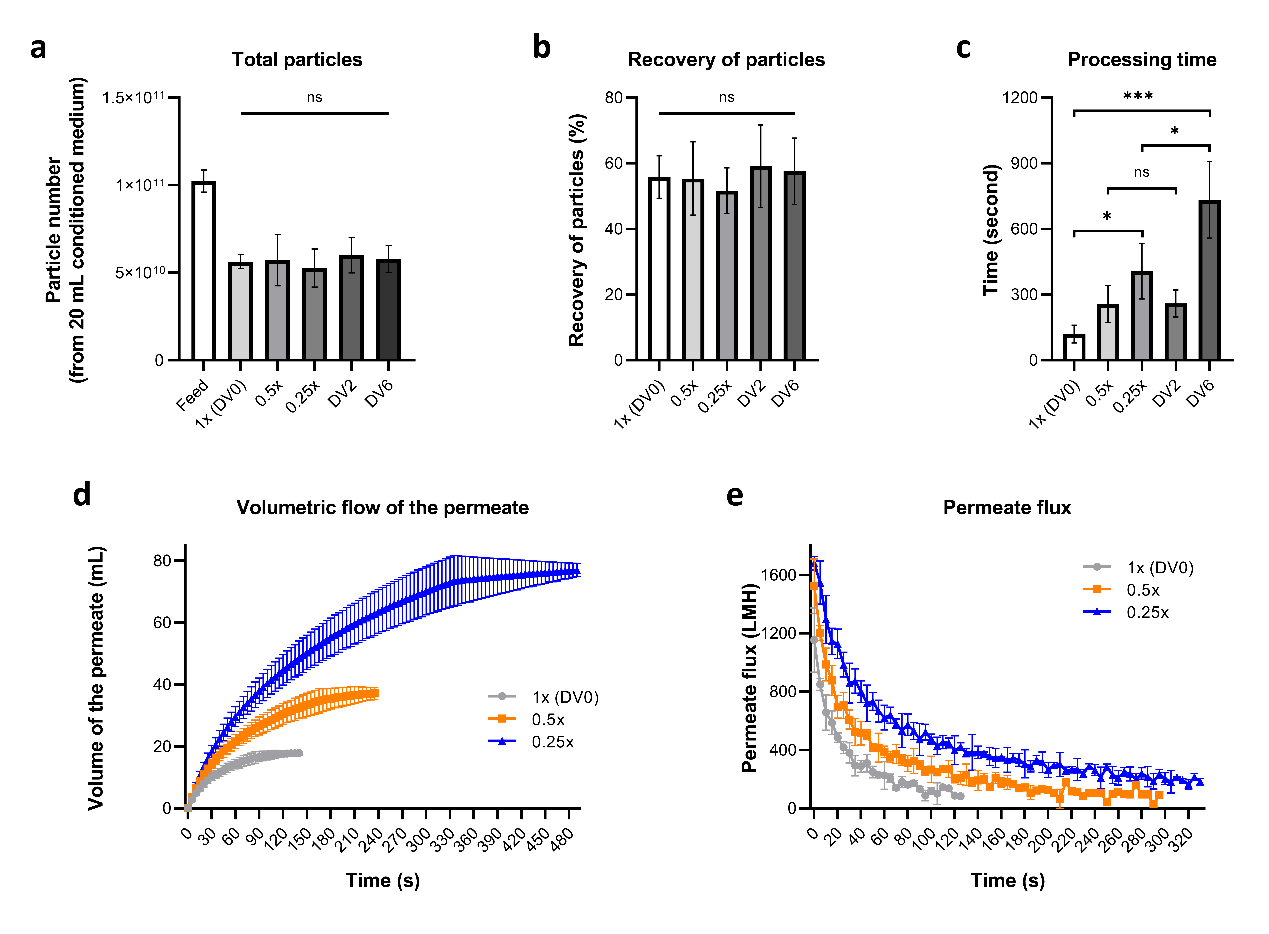


Supplementary Figure 3 The effects of direct dilution and diafiltration on particle recovery and isolation process. (a) Total particle numbers of the feed solution (CM) and the sEV preparations. (b) Recovery of particles from CM. (c) Processing time of the isolation process. (d) Volumetric flow rate of the permeate during the ultrafiltration. (e) Permeate flux during the ultrafiltration.

### Percentage of the Original Feed Volume being Replaced

**0.5x group:**

$$X_{total}=\frac{Volume reduction}{Original volume}=\frac{40-2}{40}=95\%$$

**0.25x group:**

$$X_{total}=\frac{Volume reduction}{Original volume}=\frac{80-2}{80}=97.5\%$$

**DV2 group:**

$$X_{total}=X_{pre-concentration}+X_{diafiltration}+X_{final-concentration}$$

$$X_{total}=\left( \frac{10}{20} \right)^{1}+\left[ \left( \frac{10}{20} \right)^{2}+\left( \frac{10}{20} \right)^{3} \right]+\left( \frac{10}{20} \right)^{3}\times\frac{8}{10}=97.5\%$$

**DV6 group:**

$$X_{total}=X_{pre-concentration}+X_{diafiltration}+X_{final-concentration}$$

$$X_{total}=\left( \frac{10}{20} \right)^{1}+\left[ \left( \frac{10}{20} \right)^{2}+\left( \frac{10}{20} \right)^{3}+\left( \frac{10}{20} \right)^{4}+\left( \frac{10}{20} \right)^{5}+\left( \frac{10}{20} \right)^{6}+\left( \frac{10}{20} \right)^{7} \right]+\left( \frac{10}{20} \right)^{7}\times\frac{8}{10}=99.84\%$$

where $X_{total}$ is the total removal of original feed volume in %, $X_{pre-concentration}$ is the removal of original feed volume at pre-concentration step in %, $X_{diafiltration}$ is the removal of original feed volume at diafiltration step in %, $X_{final-concentration}$ is the removal of original feed volume at final concentration step in %.
